# Prevalence of Internet Addiction and Associated Factors Among Medical Students From Mashhad, Iran in 2013

**DOI:** 10.5812/ircmj.17256

**Published:** 2014-05-05

**Authors:** Maryam Salehi, Mina Norozi Khalili, Seyed Kaveh Hojjat, Mahta Salehi, Ali Danesh

**Affiliations:** 1 Department of Community Medicine, School of Medicine, Mashhad University of Medical Sciences, Mashhad, IR Iran; 2 Research Center for Patient Safety, School of Medicine, Mashhad University of Medical Sciences, Mashhad, IR Iran; 3 Department of Psychiatry, North Khorasan University of Medical Sciences, Bojnurd, IR Iran; 4 Addiction and Behavioral Sciences Research Center, North Khorasan University of Medical Sciences, Bojnurd, IR Iran; 5 School of Medicine, Mashhad University of Medical Sciences, Mashhad, IR Iran

**Keywords:** Internet, Prevalence, Students

## Abstract

**Background:**

Problematic internet use is on the increase and has caused serious problems in many areas. This issue seems to be more important for medical students.

**Objectives:**

This study was designed to explore the prevalence of internet addiction and its related factors among the students of Mashhad University of Medical Sciences.

**Materials and Methods:**

A cross sectional study was conducted on 383 medical students of Mashhad in 2013. Four hundred participants were selected through two-stage stratified sampling method proportional to the number of students in each stage of education. Data Collection was done through using the Chen Internet Addiction Scale (CIAS) and a checklist of demographic details and characteristics of internet usage behavior.

**Results:**

It was found that 2.1% of the studied population were at risk and 5.2% were addicted users. Chatting with new people, communicating with friends and families, and playing games were the most popular activities in these groups. The factors related to internet addiction included: male sex, stage of education, daily time spent on using internet, most frequent time of internet use, monthly cost of use, and tea consumption.

**Conclusions:**

Although our study showed the prevalence of internet addiction was not more than other populations and universities, since the prevalence of internet addiction is rapidly increasing worldwide, this population might also be at risk of addiction. Thus, focusing on related factors can help us in designing more effective interventions and treatments for this susceptible group.

## 1. Background

Internet usage has been rapidly increasing worldwide. As of 2002, there were about 665 million users around the world. In Iran, there was a 3100% increase in the number of internet users between 2002 and 2006, and at present this number reaches to above 11.5 million users (1), while internet usage rate has increased 2500% from 2000 to 2010 in Arabic-speaking countries and 281% in English-speaking countries (2). Despite many potential benefits, numerous problems such as exposure to inappropriate images and content, absence of privacy and addiction to internet have been reported as a result of this increasing usage (1). Young believes that the term "addiction" can be used for internet users, since symptoms of internet addiction is comparable to the symptoms of addiction to nicotine, alcohol or drugs. Similar to other addictions, dependency is the core of internet addiction, which is defined with the presence of factors such as withdrawal syndrome, tolerance, impulsive usage and inability to control the usage (1). The term ‘internet addiction’ was first introduced by Dr. Ivan Goldberg in 1995 to describe ‘pathological and compulsive use of internet’. Griffith categorized this term as a subgroup of behavioral addictions (3). Several diagnostic criteria have been proposed and evaluated which were summarized by Buyn and colleagues (4). In addition, various psychological measures are available to assess the internet addiction which include: Young Internet Addiction Test, Problematic Internet Use Questionnaire (PIUQ), Compulsive Internet Use Scale (CIUS) (4), and Chen Internet Addiction Scale (CIAS) (5). Socio-cultural factors (such as demographic factors, ease of access, and popularity of the internet), biological propensity (such as genetic factors, unusual neuro-chemical processes), mental predisposition (such as personal characteristics, negative influences), and internet-specific characteristics predispose individuals to use internet excessively (4). As Chen and colleagues argue (2003), those who manifest addictive behaviors, are more likely to have health, socio-economic, and behavioral problems (4). There are a wide range of reports on the prevalence rate of internet addiction (0.3% to 38%) (6). Young estimated that about 5-10% of internet users were addicted to it (1). According to reports by Lejoyeux and Weinstein, the prevalence rate of internet addiction in the United States and Europe ranged from 1.5 to 8.2% (4). University students are greatly susceptible to internet addiction due to many reasons as follows:

1. University campuses provide easy and unlimited access to internet;
2. The young students experience freedom and relief out of the parental control for the first time in their lives;
3. Finding new friends is often done through internet;
4. Students encounter serious problems in the university settings;
5. The urge for usage of the modern technologies is much stronger in the youth than any other age groups;
6. The virtual atmosphere of internet lures students out of the pressure of doing university tasks and homeworks and taking exams.

Former studies estimated that 3-13% of all university students are internet addicts (5). In 2003, a research on 1360 freshmen at Taiwan University, using Chen Internet Addiction Scale (CIAS), estimated that 17.9% of them were addicted to internet (7). In the study namely “Internet Addiction and Modeling Its Risk Factors among Medical Student of Arak, Iran University”, using Young questionnaire, the prevalence of internet addiction was estimated to be 10.8%. In this study it was found that factors of age under 20 years, male sex, and using chat rooms were the most important predictors of internet addiction among students (8).

## 2. Objectives

Since young adults are considered susceptible to internet addiction, and also because of easy and quick access of students of medical sciences to internet in medical universities, and also because negligence towards this issue would cause personal, social, and educational difficulties, we decided to determine the extent of this problem and its related factors among medical students. The results of our study can help in preventing this problem in the future and designing proper interventional studies.

## 3. Materials and Methods

This cross sectional study was conducted on medical students in Mashhad, Iran during academic year 2012-2013. The sample size was estimated based on the formula for estimating prevalence. According to the prevalence of internet addiction in two previous studies (using the same questionnaire) (1, 7), considering the prevalence of 10%, α = 0.05 and precision 0.03, sample size was calculated to be 400. After the project was approved, 400 members of the target population were chosen through two stage sampling. Medical students were stratified according to the stage of education (basic sciences, physiopathology, extern and intern). Then, the required number of participants was selected by convenience sampling from each group proportionate to the number of students in each group. Students were only enrolled only after providing informed consent to participate in the study. All the participants should have used internet over the past three months prior to the study. They were assured that the questionnaires are anonymous and the study data are strictly confidential. Chen internet addiction scale (CIAS) and a checklist were used to collect the data and information. Farsi-language translation of the CIAS consists of 26 items and 5 subscales. CIAS was designed by Chen and colleagues in 2003 to assess internet addiction (5). The items were ordered according to four Likert scales:

1. strongly disagree,
2. somewhat disagree,
3. somewhat agree, and
4. strongly agree.

The score range were between 26 and 104 and a higher score indicated higher severity of internet addiction (26-63 shows normal use, 64-67 indicates at risk use and need for screening and 68-104 indicates internet addiction). Ramazani and colleagues (2012) validated this questionnaire among Iranian medical students (1). The results of this questionnaire are useful for describing a total index, two scales of ‘main symptoms of internet addiction’ (IA-Sym), ‘internet addiction-related problems’ (IA-RP), and five subscales of compulsive symptoms (Com), withdrawal (Wit), tolerance symptoms (Tol), interpersonal health problems (IH) and time management difficulties (TM). In the original study, Chen and colleagues estimated Cronbach's alpha of scale and subscales of CIAS questionnaire to range from 0.79 to 0.93. In 2005, a similar study by Ku et al. determinated Cronbach's alpha to be 0.94 (9). Ramazani and colleagues also had reported the value of Cronbach's alpha for subscales which was between 0.67 and 0.85. Also, in this study the convergence co-efficient of r = 0.85 with P < 0.001 between CIAS and IAT (Young internet addiction questionnaire) indicated high convergence validity of this questionnaire (1). Thus, previous studies have confirmed a high degree of reliability and validity of this questionnaire. In our study, the dependent variable was internet addiction. Independent and background variables in this study included: age, gender, location of residence, marital status, stage of education, monthly cost of internet services, predominant time of internet use, length of internet use, type of internet activity and tea, coffee and cigarette consumption. The required number of questionnaires were filled out by medical students, data were collected and then analyzed by SPSS version 11.5. First, characteristics of each group were described using central and dispersion measures and were presented by tables and charts. Then, in order to compare qualitative variables among groups, Chi-square test was used. For quantitative variables, normality of data was assessed by K-S test. T-test was used for comparing means between two independent groups with normal distribution. In case of non-normal distribution, the equivalent non-parametric test (Mann-Whitney) was used. For all the analyses, level of significance was set at P < 0.05.

## 4. Results

Out of 400 distributed questionnaires, 383 students participated in our study, from whom 149 (38.9%) were male, and 234 (61.1%) were female. The mean age of the participants was 21.79 ± 2.42 (range = 17-30). Table 1 shows the demographic characteristics and other factors related to internet use among the participants. Mean length of internet use was 1.87 ± 1.72 hours per day and its range was between zero to ten hours.

**Table 1.** Demographic Characteristics and other Factors Related to Internet Use Among Medical Students of Mashhad University in 2013^a^

Variables	
**Gender**	
Male	149 (38.9)
Female	234 (61.1)
**Age range, y**	17 – 30 (21.79 ± 2.42)
**Stage of education**	
Basic sciences	152 (39.7)
Physiopathology	54 (14.1)
Extern	91 (23.8)
Intern	86 (22.5)
**Marital status**	
Single	346 (90.3)
Married	37 (9.7)
**Location of residence**	
With parent or spouse	245 (64)
Dormitory	103 (26.9)
Other	35 (9.1)
**Smoking**	
Yes	17 (4.5)
No	366 (95.5)
**Predominant time of internet use**	
Morning to evening	239 (62.7)
Night and midnight	143 (37.3)
Daily time range of Internet use	0 – 10 (1.87 ± 1.72)
**Estimated monthly cost of internet use by subject**	
Very low to low	202 (52.7)
Intermediate	151 (39.4)
High to very high	20 (5.2)
**Main location of internet use**	
House and dormitory	354 (92.6)
Other (university)	28 (7.4)
**Speed of internet**	
Intermediate	116 (30.3)
High	266 (69.7)
Consumption of tea (cup)	2.6 ± 2.2
Consumption of coffee (cup)	0.45 ± 0.86

^a^ Data presentation are means ± SD or No. (%).

All 383 participants used internet for various purposes: 11 people (2.9%) used internet for playing games; 129 people (33.7%) for downloading film and music; 24 people (6.3%) for chatting with new people; 153 people (39.9%) for scientific search; 134 people (35%) for communicating with friends and families; 207 people (54%) for checking email; 22 people (5.7%) for internet shopping; 96 people (25.1%) for reading news; and finally, 21 people (5.5%) for writing weblogs. Table 2 shows mean, standard deviation, and range of scores for scales and subscales of CIAS questionnaire in this study. According to CIAS questionnaire and considering the cut points of 63, 67, 92.7 % of the studied populations were not addicted to internet but 2.1% were at risk and 5.2% were internet-addict, the last two groups were considered as problematic groups (Table 3).

**Table 2.** The prevalence of internet Addiction (According to Defined Scores) Among Students of Mashhad University of Medical Sciences in 2013

The Type of Addiction (Due to Range of Defined Scores)	No. (%)
**No addicted to internet (26 - 63)**	355 (92.7)
**At risk and need to screening (64-67)**	8 (2.1)
**Internet addicted (at or above 68)**	20 (5.2)

**Table 3.** Mean, Standard Deviation, and the Range of Scores for Scale and Subscales of Chen Internet Addiction Questionnaire (CIAS)

Variables	Range, y	Mean ± SD
**Compulsive symptoms (Com)**	5-20	7.75 ± 2.95
**Withdrawal symptoms (Wit)**	5-20	9.86 ± 3.58
**Tolerance symptoms (Tol)**	4-17	7.54 ± 2.55
**Interpersonal health problem (IH)**	7-28	10.43 ± 3.83
**Time management (TM)**	5-20	7.72 ± 3.08
**Main symptoms of internet addiction (IA-Sym)**	14-53	25.17 ± 7.93
**Internet addiction-related problems (IA-RP)**	12-48	18.15 ± 6.55
**Internet addiction**	26-101	43.32 ± 13.6

The results revealed a significant relationship between sex and the pattern of internet use, as 72% of problematic-user group and 36% of normal group were male (P < 0.001). There was a significant relationship between the stage of education and the pattern of internet use, as students of basic sciences formed the greatest portion of the problematic group (P = 0.04). Regarding the mean age and marital status, no significant differences were observed between two groups (Table 4).

**Table 4.** The Results of Analytic Tests to Compare Demographic Characteristics and Other Factors Related to the Internet Use Between Normal and Problematic Groups^a^

Variables	Normal Internet Users	Problematic Internet Users	P value
**Age, y**	21.83 ± 2.4	21.26 ± 2.75	0.19
**Gender**			< 0.001
Male	128 (36)	20 (72)	
Female	226 (64)	8 (28)	
**Stage of education**			0.04
Basic sciences	135 (38)	17 (61)	
Physiopathology	52 (14.5)	2 (7)	
Extern	89 (25)	2 (7)	
Intern	79 (22.5)	7 (25)	
**Marital status**			0.5
Single	319 (90)	27 (96)	
Married	36 (10)	1 (4)	
**Location of residence**			0.78
With parent or spouse	228 (64)	17 (61)	
Dormitory	33 (9.5)	2 (7)	
Other	94 (26.5)	9 (32)	
**Daily time of internet use, h**	1.7 ± 1.54	3.92 ± 2.39	< 0.001
**Predominant time of internet use**			0.02
Morning to evening	227 (64)	12 (43)	-
Night and midnight	127 (36)	16 (57)	-
**Main location of internet use**			
House or dormitory	291 (82.2)	27 (96.4)	0.06
Other (university)	63 (17.8)	1 (3.6)	
**Estimated monthly cost of internet use by subject**			< 0.001
Very low to low	196 (56.6)	6 (22.2)	
Intermediate	136 (39.4)	15 (55.6)	
High to very high	14 (4)	6 (22.2)	
**Speed of utilized internet**			0.28
Intermediate	110 (31)	6 (21.5)	
High	244 (69)	22 (78.5)	
**Smoking**			0.81
Yes	16 (4.5)	1 (3.5)	
No	339 (95.5)	27 (96.5)	
**Daily tea use (cups)**	2.5 ± 2.13	3.64 ± 2.64	0.01
**Daily coffee use (cup)**	0.46 ± 0.88	0.43 ± 0.57	0.64

^a^ Data is presented as mean ± SD or No. (%).

Mean length of daily internet use, predominant time of use, and mean monthly cost of internet services were significantly different between two groups. So, in the group with normal use, mean daily internet use was 1.7 ± 1.54 hours per day, while in the problematic group, it was 3.92 ± 2.39 (P < 0.001) and the latter group used internet during the night and midnight much more frequently than the normal group (P = 0.02). Also, problematic users spend more on internet than normal users (P < 0.001). Mean daily tea consumption was significantly different among these groups so that problematic users drank more tea than the normal group. However, drinking coffee was not different between these groups. Smoking cigarette was not significantly different among the groups (P = 0.81) (Table 4).

Relative frequency of each type of internet activity is shown in Table 5, where the most and the least frequent types of them were checking emails and playing games, respectively. Using the proper statistical tests, the distribution of frequency of playing games, chatting with new people and communicating to friends and families were found to be more frequent in the problematic group compared to the normal group and these differences were statistically significant. In contrast, downloading films and music, scientific search, checking e-mails, internet shopping, reading news, and writing weblogs were not significantly different between the two groups.

**Table 5.** The Results of Analytic Tests to Compare Frequency of Internet Activities Between Normal and Problematic Groups ^a^

Internet Activities	Normal Internet Users	Problematic Internet Users	Total	P value
**Playing games**				0.04
Yes	8 (2.3)	3 (10.7)	11 (2.9)	
No	347 (97.7)	25 (89.3)		
**Downloading films & music**				0.28
Yes	117 (33)	12 (42.9)	129 (33.7)	
No	238 (67)	16 (57.1)		
**Chatting with new people**				< 0.001
Yes	15 (4.2)	9 (32.1)	24 (6.3)	
No	340 (95.8)	19 (67.9)		
**Scientific search**				0.09
Yes	146 (41.1)	7 (25)	153 (39.9)	
No	209 (58.9)	21 (75)		
**Communicating to friends & families **				0.003
Yes	117 (33.1)	17 (60.7)	134 (35)	
No	237 (66.9)	11 (39.3)		
**Checking email**				0.95
Yes	192 (54.1)	15 (53.6)	207 (54)	
No	163 (45.9)	13 (46.4)		
**Shopping**				0.6
Yes	20 (5.6)	2 (7.1)	22 (5.7)	
No	335 (94.4)	26 (92.9)		
**Reading news**				0.06
Yes	93 (26.2)	3 (10.7)	96 (25.1)	
No	262 (73.8)	25 (89.3)		
**Writing weblog**				0.057
Yes	17 (4.8)	4 (14.3)	21 (5.5)	
No	338 (95.2)	24 (85.7)		

^a^ Data are presented as No. (%).

## 5. Discussion

This study showed that 2.1% of total number of participants were at risk and 5.2% were addicted users, so 7.3 % of all participants were considered problematic users. In a study conducted by Deng and colleagues, it was also found that the prevalence of this disorder was 5.52% among students which is consistent with our own results. Similarly, Ramazani and colleagues found the total prevalence of 3% for Iranian medical students (1). Similar study was conducted among students of Turkish University of Medical Sciences showing the prevalence of internet addiction is 24 (10.3%) among nursing students, 7 (9.9%) among midwifery students, 5 (9.1%) among medical rescue students and 42 (19.6%) among physiotherapy students (10, 11). It must be noted that comparing these studies is a difficult task because of differences in study populations, applied tools and differences in social and cultural contexts. The participants of this study stated the main purposes of using internet as the following (in order of importance): checking emails, scientific search, communicating to friends and families, downloading films and music, chatting with new people, internet shopping, blogging, and finally playing games. In this study, the most frequent uses of internet among problematic internet users were chatting with new people, communicating to friends and families and online gaming. The first two activities are the most important activities related to internet dependency which is consistent with the fact confirmed by other researches that addicted users mostly prefer chat rooms (1, 3, 8, 10, 12, 13). Similar to the most of the other studies, this study showed that there was no significant relation between internet dependency and use of internet for scientific search; this finding was consistent with other studies (14). In contrast, in a survey titled "Internet Addiction and the Related Factors in Residents of Zone 2 of Western Tehran", which surveyed people 15 to 39 years of age, Dargahi and colleagues proved that internet use was related to scientific activities (15); this contradiction was mostly attributed to the differences in study populations. Similar to the previous studies, results of this study also indicated that there was a significant relationship between playing games and internet addiction (12, 16). In this study, it was found that mean age of participants was not significantly different between two groups which is consistent with the results of studies conducted by Bernardi and colleagues (17) and Mohammad Beigi and colleagues on the students of Arak University of Medical Sciences. However, most of the previous researchers had concluded that there was a significant relationship between severity of addiction and age, so, younger people are at higher risk for internet addiction disorder (7, 8, 15, 18-20). Maybe the reason for this contradiction was that the studied population of the prior studies had a greater range of age. According to this study, internet addiction was more common in males which is consistent with previous researches (3, 7, 8, 12, 21-24). In the study conducted by Ikenna Adiele and Wole Olatokun on adolescents, male to female ratio was approximately 3:1 for internet-addict subjects (25).

According to this study, problematic internet users spent longer hours using internet than normal users, which was consistent with previous studies (13, 23). Wasting time is one of the greatest causes of poor functioning among addicted users.

 Our study suggested a significant relationship between the stage of education and internet addiction. Our study discovered no relation between marital status and internet addiction. Nevertheless, such a relation was found in the most of the previous studies which found that internet addiction was more common among single rather than the married subjects (15). In our study, the main place of internet use was not significantly different between the study groups. Studies found that the location of internet access is a potential risk factor of internet addiction (12, 22, 26, 27). Our results showed that problematic users mostly tended to use the internet at night and midnight. Among the medical students, use of the internet in the night and midnight causes social, academic or occupational problems, which even might exacerbate internet addiction in this group (28). One of the strengths of this study was that the participants were chosen from all stages of education and also related factors of internet addiction were evaluated. However, there are some limitations to our study. First, no interview was conducted to confirm diagnosis of internet addiction. Second, we only tried to establish a relation between internet addiction and potential risk factors without being able to prove any cause and effect relation between them. Finally, some refused to fill out the questionnaires which could negatively affect the strength of our study. Although our study showed that the prevalence of internet addiction was not more than other populations and universities, as the prevalence of internet addiction is increasing rapidly worldwide, the studied population might also be at increased risk of internet addiction. Thus, focusing on related and causing factors can help us design more effective interventions and treatments for this susceptible group. Finally, we suggest that further studies be conducted by interviewing the subjects to determinate the causes and factors related to internet addiction among students.
